# A giant spinal schwannoma at the C1-C2 level: A case report

**DOI:** 10.1097/MD.0000000000041258

**Published:** 2025-01-03

**Authors:** Xiaodong Wu, Dazhi Li, Lu Zhang, Yanming Li

**Affiliations:** a Jining Medical University, Jining, China; b Affiliated Hospital of Jining Medical University, Jining, China

**Keywords:** C1-C2, giant spinal schwannoma, vertebral artery

## Abstract

**Rationale:**

Giant spinal schwannomas located at the C1-C2 level pose significant surgical challenges because of their proximity to the brainstem, cervical spinal cord, and vertebral arteries. This case provides insight into the surgical management of giant spinal schwannomas at the C1-C2 level.

**Patient Concerns:**

A 40-year-old female presented with a 2-year history of progressive limb numbness, weakness, and gait instability. She reported a sensation of stepping on cotton and tightness in the chest and abdomen with worsening symptoms over time.

**Diagnoses:**

Physical examination revealed limited cervical spine movement, increased muscle tone in the limbs, and Hoffmann and Babinski signs. Imaging, including CT, CTA and MRI, identified a large schwannoma at the C1-C2 level, with compression of the spinal cord and proximity to the vertebral artery.

**Interventions:**

The patient underwent surgical excision of the tumor using full laminectomy approach under intravenous anesthesia.

**Outcomes:**

The surgery was successfully completed, and the patient’s neurological symptoms, including numbness and weakness, were significantly relieved.

**Conclusion:**

Strict enucleation within the capsule can effectively protect the vertebral artery and nearby nerves. When functional nerve roots or the spinal cord are involved, leaving the residual capsule may be safer than risking permanent deficits.

## 1. Introduction

Spinal schwannomas account for approximately 25% of all spinal tumors.^[1]^ These tumors are typically small, benign, and isolated, making surgical resection straightforward with good outcomes.^[2]^ However, giant schwannomas, particularly at the C1-C2 level, pose significant challenges due to their proximity to the brainstem, cervical spinal cord, and vertebral arteries, increasing the risk of complications such as hemorrhage, respiratory failure, or vascular injury during surgery.^[3,4]^

## 2. Case report

**Figure 1.**
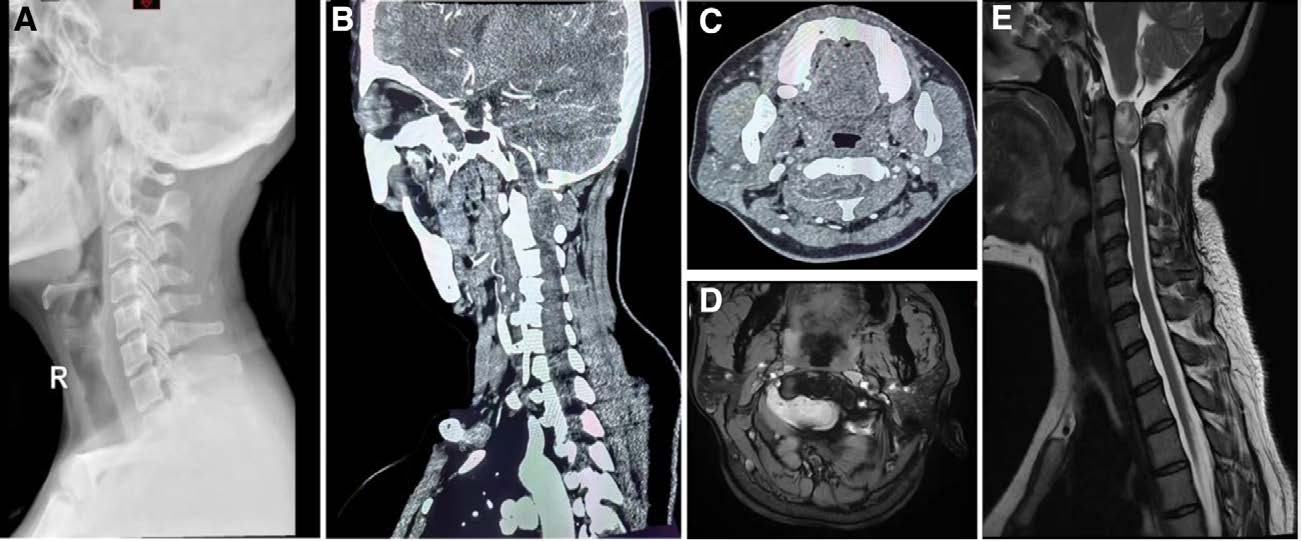
Imaging findings on first admission.

A 40-year-old female was admitted to the hospital with numbness and weakness in her limbs and unsteady walking for 2 years. Over the past 2 years, the symptoms gradually worsened, and there was a feeling of stepping on cotton under the feet and a feeling of restraint in the chest and abdomen. Physical examination limited movement of the cervical spine, posterior neck and scapular tenderness, increased muscle tone in limbs, bilateral flexion and extension of the elbow, flexion and extension of the wrist, and intrinsic muscle strength of the hand level 4, bilateral knee flexion and extension, plantar flexion, and dorsal foot extensor muscle strength level 4, hyposensation in the limbs, and decreased sensation in the trunk from the sternal angle to the distance. Bilateral Hoffmann and Babinski signs were positive, with hyperreflexia of knee reflex (+++) and Achilles tendon reflex (+++). A plain film showed there was no obvious abnormality in the bone quality of the cervical vertebra (Fig. 1A), However, computed tomography and computed tomography angiography showed a space-occupying lesion in the right C1-C2 foraminal region (20mm × 43mm × 30mm), with indistinct demarcation from the V4 segment of the vertebral artery (Fig. 1B and C). Magnetic resonance imaging (MRI) revealed compression and deformation of the spinal cord adjacent to the lesion. (Fig. 1D and E). Regrettably, we were unable to perform a preoperative electromyography examination to assess the patient’s nerve damage.

The patient presented with a large tumor closely associated with the spinal cord and vertebral artery, with the possibility of extension into the dura. This anatomical complexity posed a significant risk of respiratory and cardiac arrest, as well as paralysis, during surgery. After a thorough multidisciplinary discussion, the decision was made to proceed with cervical spine tumor resection, which was performed on January 3, 2024, under intravenous anesthesia.

**Figure 2.**
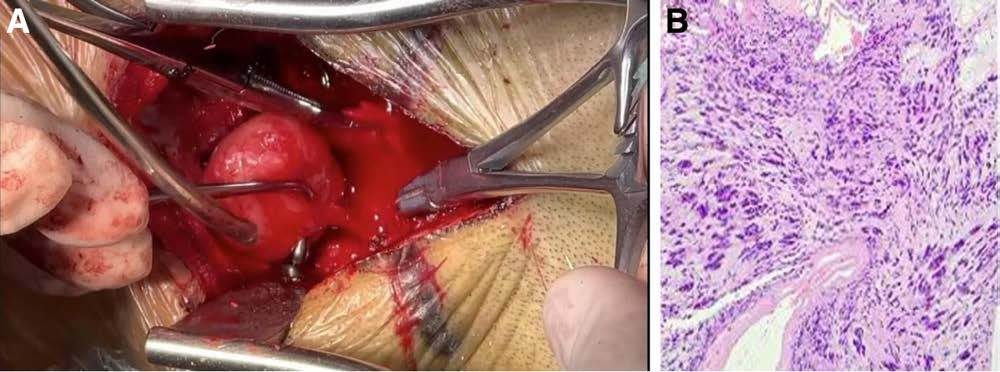
(A) Intraoperative image of the tumor. (B) Postoperative pathology results.

Following successful induction of anesthesia, skull traction was applied, and precise dissection was performed in the layers under intraoperative X-ray guidance. The posterior arch of the atlas was resected, followed by the removal of the right half of the lamina and the spinous

process of the axis. Due to the significant thinning of the pedicle caused by the tumor’s mass effect, internal fixation could not be performed. Intraoperatively, the tumor was found to be entirely located in the epidural space, situated anterolaterally to the upper cervical cord and extending laterally into the right nerve root canal. Complete excision of the tumor was achieved with capsule preservation. The dura mater remained intact during the procedure. A portion of the greater occipital nerve, which was closely adherent to the tumor, was also resected (Fig.2A). Intraoperative neurophysiological monitoring of the motor and sensory pathways revealed no significant changes compared with preoperative baselines. The incision was sutured in layers, and a posterior neck collar was applied for immobilization. Postoperative pathology confirmed the diagnosis of schwannoma, with immunohistochemistry showing S-100 positivity in the tumor cells (Fig. 2B).

**Figure 3.**
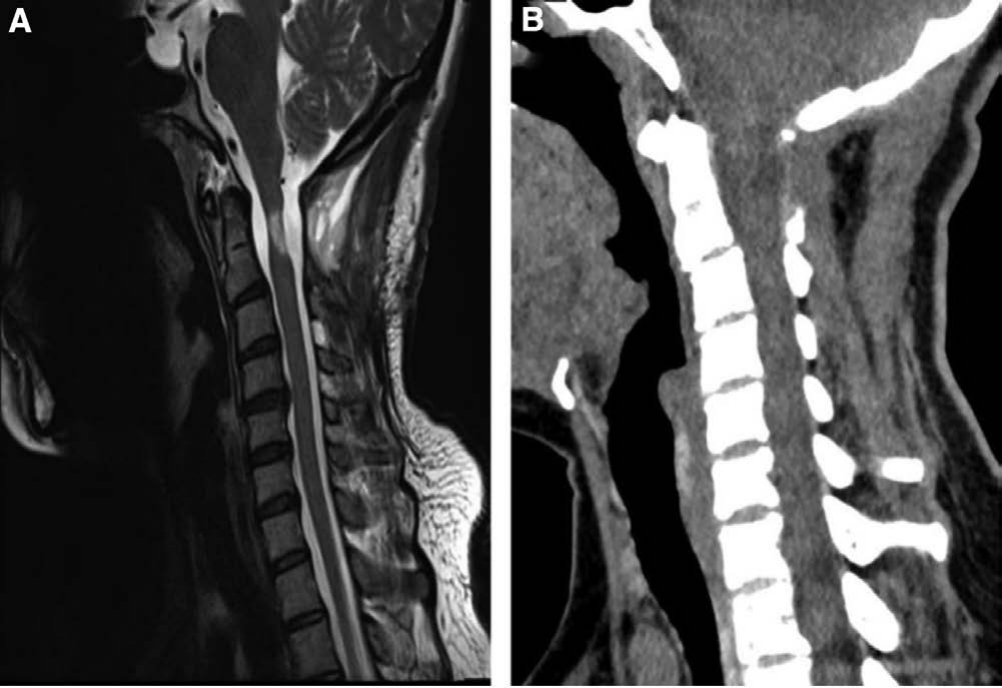
(A) MRI on the fifth day after surgery. (B) CT after 3 months of follow-up.

After the operation, the patient’s symptoms of numbness, weakness, and hypesthesia were significantly relieved. On the fifth day after surgery, MRI confirmed the resolution of spinal cord compression (Fig. 3A). At the 3-month follow-up, the patient remained asymptomatic and CT scans showed no evidence of vertebral fracture or instability (Fig. 3B).

## 3. Discussion

Schwannomas originate from Schwann cells and are encapsulated early in their formation.^[5]^ As the tumor grows, surrounding soft tissues, including fibers and connective tissue, form a pseudo-capsule around it.^[6]^ These capsule structures are critical for ensuring the safe surgical resection of the tumor. In complex cases involving the vertebral artery, the artery may pass behind or above the tumor, and attempting to excise the tumor from outside the capsule increases the risk of damaging the artery. Therefore, maintaining the integrity of the tumor capsule is essential when managing large spinal schwannomas. Even when preoperative imaging shows close proximity to the vertebral artery, intracapsular resection is often feasible due to a small gap between the tumor and the artery.^[7]^ This approach allows for complete tumor removal while minimizing the risk of arterial injury.

Selection of the surgical approach is also of paramount importance in cases of giant schwannomas. The entire lamina approach offers the advantages of a wide surgical field and ample operating space. However, it involves the removal of muscles, ligaments, spinous processes, and lamina, which may lead to postoperative spinal instability.^[8]^ In contrast, the hemilamina approach minimizes damage to the supraspinous ligament, interspinous ligament, and lamina, reducing complications associated with the entire lamina approach. However, the surgical field is limited to the side of the laminectomy, which significantly reduces the operative space and increases the risks of nerve injury, incomplete tumor resection, and insufficient hemostasis. In this case, due to the tumor growing predominantly on the right side and considering the critical role of the axis in maintaining the mobility and stability of the cervical spine, we initially opted for a hemilaminectomy. After removing the right hemilamina, the surgical exposure was still unsatisfactory because of the tumor’s large size. Subsequently, the spinous process of the axis was further removed, which ultimately allowed the surgery to proceed successfully.

The delicate balance between maximizing tumor resection and minimizing neurological injury is a persistent challenge in the surgical treatment of giant schwannomas. When conditions allow, gross total resection is considered curative.^[9]^ However, in cases where the tumor is closely related with the nerve roots and spinal cord, performing a gross total resection involving the removal of the affected nerve roots may lead to sensory numbness, partial motor weakness, or even paralysis.^[10,11]^ On the other hand, subtotal resection may increase the risk of tumor recurrence, but due to the slow growth of these tumors, even in cases of recurrence, subtotal resection is often sufficient to provide long-term symptom relief.^[12]^ Some scholars suggest that sensory nerve roots adhered to the tumor can be excised, but when the tumor involves functional nerve roots or the spinal cord, leaving a residual capsule is preferable to causing neurological deficits.^[10–12]^

## 4. Conclusion

The surgical treatment of giant spinal schwannomas, especially at the C1-C2 level, is complex due to their proximity to vital neurovascular structures. Strict enucleation within the capsule can effectively protect the vertebral artery and nearby nerves. While gross total resection is often curative, preserving neurological function is crucial, particularly when functional nerve roots or the spinal cord are involved. In such cases, leaving the residual capsule may be safer than risking permanent deficits. Successful management requires balancing complete resection with preserving the patient’s quality of life.

## Acknowledgments

The authors would like to thank our department colleagues and these patients for their dedication, and those patients or their next of kin had signed the informed consent forms.

## Author contributions

**Conceptualization:** Dazhi Li.

**Writing –original draft:** Xiaodong Wu.

**Writing – review & editing:** Yanming Li, Lu Zhang.
